# Efficacy and safety of normobaric hyperoxia as an adjunct to endovascular thrombectomy in acute ischemic stroke: A systematic review and meta-analysis of randomized controlled trials

**DOI:** 10.1007/s11239-025-03193-0

**Published:** 2025-11-08

**Authors:** Omar Kassar, Rashad G. Mohamed, Khalid Sarhan, Muataz Kashbour, Maaly Ali Abuhlaiga, Haneen Sabet, Omar Elawwad, Obai Yousef, Moaz Elsayed Abouelmagd

**Affiliations:** 1 Faculty of Medicine, Alexandria University, Alexandria, 21527 Egypt; 2 Mansoura Manchester Program for Medical Education, Faculty of Medicine, Mansoura University, Mansoura, Egypt; 3 Faculty of Medicine, Mansoura University, Mansoura, Egypt; 4 Dignostic Radiology Department, National Cancer Institute, Misrata, Libya; 5 Nephrology Department, Misrata Medical Center, Misrata, Libya; 6 Faculty of Medicine, South Valley University, Qena, Egypt; 7 Medical Research Group of Egypt, Neida Academy, Arlington, MA USA; 8 Department of Neurosurgery, Tishreen University Hospital, Latakia, Syria; 9 Faculty of Medicine, Cairo University, Cairo, Egypt

**Keywords:** Brain oxygenation, Cerebral ischemia, Endovascular treatment, Meta-analysis, Normobaric hyperoxia, Stroke

## Abstract

Endovascular thrombectomy (EVT) is the standard of care in acute ischemic stroke (AIS), yet functional outcomes remain suboptimal. Normobaric hyperoxia (NBHO) is a potential neuroprotective strategy. This study is the first systematic review and meta-analysis to assess NBHO as a potential neuroprotective adjunctive to improve outcomes in EVT-treated patients. A comprehensive search of electronic databases, including PubMed, Scopus, Cochrane, and Web of Science, was performed in February 2025. The inclusion criteria targeted randomized controlled trials (RCTs) comparing NBHO and EVT to EVT alone or with sham oxygen therapy. Statistical analyses were performed using RevMan software. Four RCTs comprising 648 patients with AIS due to large vessel occlusion in the anterior circulation were included in the study. For the primary efficacy endpoint of excellent functional outcome, defined as the number of patients who had a Modified Rankin Scale (mRS) score of ≤ 1 at 90 days, the overall odds ratio with a subgroup based on the oxygen delivery duration at 2, 4, and 6 h was in favor of the NBHO group compared to the control (OR = 1.66, 95% CI [1.13, 2.45], *P* = 0.01, I2 = 0%). The subgroup of 4-hour oxygen delivery duration was the only significant subgroup (OR = 1.6, 95% CI [1.01, 2.51], *P* = 0.04). Safety outcomes showed no significant differences between the NBHO group and the control group across all reported measures. NBHO as an adjunct to EVT appears to be effective and safe. A 4-hour duration was found to be the most effective. Further RCTs are needed to confirm our results and establish the optimal treatment protocol.

**Graphical abstract:**

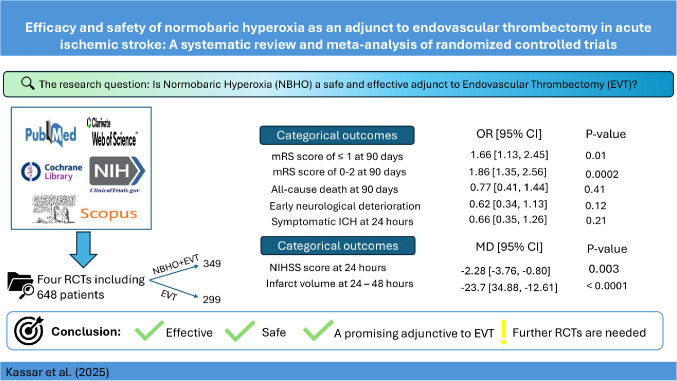

**Supplementary Information:**

The online version contains supplementary material available at 10.1007/s11239-025-03193-0.

## Introduction

Stroke, particularly the ischemic subtype, remains a leading global cause of disability. According to the World Stroke Organization, 12.2 million new strokes occur per year, and around 101 million are living with stroke-related consequences [1]. These figures drive the continuous research efforts towards better Acute Ischemic Stroke (AIS) treatment strategies.

Currently, endovascular thrombectomy (EVT) is the most effective treatment for AIS [2]. Successful EVT rates have exceeded 80%; however, despite these improvements, relative disparities exist between successful revascularization rates and good functional outcomes, which remain below 50% [3, 4]. This may be attributable to many factors, such as the limited “salvageable ischemic penumbra” and the occurrence of reperfusion injury [5–7]. EVT is a very time-restricted treatment modality, and the ischemic penumbra can rapidly evolve, becoming part of the ischemic core before EVT is available. It is this disparity between successful EVT rates and favorable functional outcomes that highlights the need for neuroprotective approaches, along with EVT, to help “freeze the penumbra” and potentially improve patients’ functional outcomes [8, 9].

Most postulated neuroprotective approaches failed to translate into clinically effective approaches despite promising preclinical studies. This failure is thought to be due to the reduced delivery of neuroprotective agents via the damaged blood-brain barrier (BBB), among other reasons. Oxygen supplementation has been suggested to reduce injury of the ischemic penumbra, minimize tissue damage, reduce infarct volume, and enhance neural recovery [10–12]. Normobaric Hyperoxia (NBHO) -which is the continuous administration of oxygen to patients via a mask under normal atmospheric pressure- is gaining popularity due to its ease of administration across different settings, rapid onset of action, widespread availability, minimal adverse events, and low cost. However, previous clinical trials have failed to demonstrate a consistent neuroprotective effect of NBHO in ischemic stroke [13]. This was largely explained by not combining NBHO with EVT in these clinical trials [14].

To the best our knowledge, this is the first systematic review and meta-analysis aimed at systematically summarizing the available body of evidence evaluating the safety and efficacy of NBHO, with a particular focus on identifying the most effective oxygen administration duration as an adjunctive therapy for patients undergoing EVT for AIS aspiring to reduce the global stroke disability burden.

## Methods

### Protocol

We followed the Preferred Reporting Items for Systematic Reviews and Meta-Analyses (PRISMA statement) guidelines when reporting this study [15]. This manuscript was conducted in adherence to the Cochrane Handbook of Systematic Reviews of Interventions [16]. All authors worked independently at every stage of the study.

### Search strategy and data source

We conducted a comprehensive search on 16 February 2025, across the following databases: PubMed, Web of Science, Scopus, and the Cochrane Library for eligible studies. The search approach used MeSH (medical subject headings) and their synonyms. (“normobaric hyperoxia” OR “high-flow oxygen” OR “high oxygen therapy” OR “100% oxygen”) AND (“acute ischemic stroke” OR “ischemic stroke” OR “stroke” OR “cerebral ischemia” OR “brain ischemia” OR “cerebral infarction”) were the search strategy used. The search terms were combined using Boolean operators to ensure comprehensive retrieval of relevant studies. Additionally, Google Scholar results and references of included studies were manually screened for any possible studies to include.

### Eligibility criteria

Studies were selected based on predefined inclusion and exclusion criteria. Studies satisfying the following criteria were included: [1] randomized controlled trials (RCT) [2], adult patients diagnosed with acute ischemic stroke due to large-vessel occlusion (LVO) in the anterior circulation [3], normobaric hyperoxia plus endovascular treatment as the intervention group [4], endovascular treatment with or without sham oxygen as the control group. Studies were excluded if they were: [1] case reports, case series, observational studies, conference abstracts, and thesis [2], animal studies or in vitro studies [3], reviews, book chapters, theses, letters, and overlapped datasets, and [4] non-English studies.

### Screening and study selection process

Two independent authors used Rayyan for semi-automated screening of the literature search results [17]. Studies were screened in two phases; we screened the titles/abstracts for potential clinical studies in the first phase. In the second phase, we retrieved the full-text articles of the selected abstracts for further eligibility screening.

### Data extraction

Data extraction was independently performed by two authors using a standardized data extraction form developed in Microsoft Excel. Extracted data included study characteristics, such as author(s), year of publication, country, study design, clinical trial registry numbers, sample size, inclusion criteria, treatment protocol, comparator, outcome measures, and key findings of included studies. Second sheet included baseline characteristics of the included studies such as, sample size, age, sex, baseline NIHSS (National Institutes of Health Stroke Scale) stroke score, and number of patients with comorbidities such as, atrial fibrillation, hypertension, and diabetes. A third outcome sheet was specifically designed to collect the outcomes of each study. Any disagreements in data extraction were resolved by consensus or consultation with a third author.

### Risk of bias assessment

The quality of the studies included was evaluated by two independent authors using the Cochrane risk of bias assessment tool for RCTs version two (ROB2) [18]. Bias potential was evaluated across five domains, including (1) bias due to randomization process (2) bias due to deviations from intended interventions (3) Bias due to missing outcome data (4) Bias in measurement of the outcome (5) Bias in selection of the reported result. The overall judgment for each domain was classified into one of three levels: low, moderate, or high risk of bias. Conflict between authors was resolved through mutual agreement or by consulting the primary investigator.

### Outcome definition

The primary efficacy outcome was an excellent functional outcome defined as a modified Rankin Scale (mRS) score of ≤ 1 at 90 days. Additional efficacy outcomes were the mean change of NIHSS score from baseline at 24, 72 h, and 7 days, and infarct volume at 24 to 48 h. Safety outcomes were death (all-cause and stroke-related), symptomatic intracranial hemorrhage, serious adverse events, pneumonia, and early neurological deterioration.

### Data synthesis and statistical analysis

We analyzed the data in accordance with the intention- to-treat principle. Odds ratios with the corresponding 95% confidence interval (CI) were computed for dichotomous variables (e.g., mRS subgroups, early neurological improvement, death [all causes and stroke related], symptomatic ICH, pneumonia, early neurological deterioration, and serious adverse events). For continuous variables (e.g. mean mRS score at 90 days, NIHSS score at 24 h, 72 h, and 7 days, and post treatment infarct volume), the mean difference (MD) and 95% CI were computed to pool effect estimates. Wan et al. method was used to convert any outcome reported in the form of median and interquartile range (IQR) to mean and standard deviation (SD) [19]. The standard DerSimonian–Laird random-effects model was used as the lead approach, as they account for fewer assumptions. In addition, we repeated the primary analysis using he Hartung–Knapp–Sidik–Jonkman (HKSJ) adjustment as a sensitivity analysis. The evaluation of heterogeneity across studies was assessed with the application of Higgin’s index (I^2^) to quantify this heterogeneity, and the Chi-Square test was used to assess the significance of heterogeneity. A high degree of heterogeneity was considered when the I^2^ value fell within the range of 75% to 100% [20]. In the Chi-Square test, a P value of less than 0.1 was considered statistically significant. The Cochrane Collaborations Review Manager Software Package (RevMan 5.4.1) and R studio were used for this meta-analysis. We conducted subgroup analysis based on the follow-up durations and the duration of oxygen therapy. We conducted sensitivity analysis to explore the source of heterogeneity when found. To ensure correct statistical estimation and avoid double counting of patients, we assessed a potential overlap between Li 2024 and Li 2025. Despite having different NCT numbers, both studies shared some recruitment months and one center. We contacted the first author, Weili Li, who confirmed that the study populations were completely different, with no overlap at all.

### Publication bias

In accordance with Egger et al., we did not assess publication bias using Egger’s test for funnel plot asymmetry. The Egger test requires a minimum of 10 included studies to be applicable [21].

### Certainty of evidence

The certainty of the evidence for this meta-analysis was evaluated using the Grading of Recommendations, Assessment, Development, and Evaluation (GRADE) tool. This tool considers criteria such as study design, risk of bias, inconsistency, indirectness, imprecision, and publication bias. The certainty of the evidence can be rated as high, moderate, low, or very low [22].

## Results

### Study selection

**Fig. 1:**
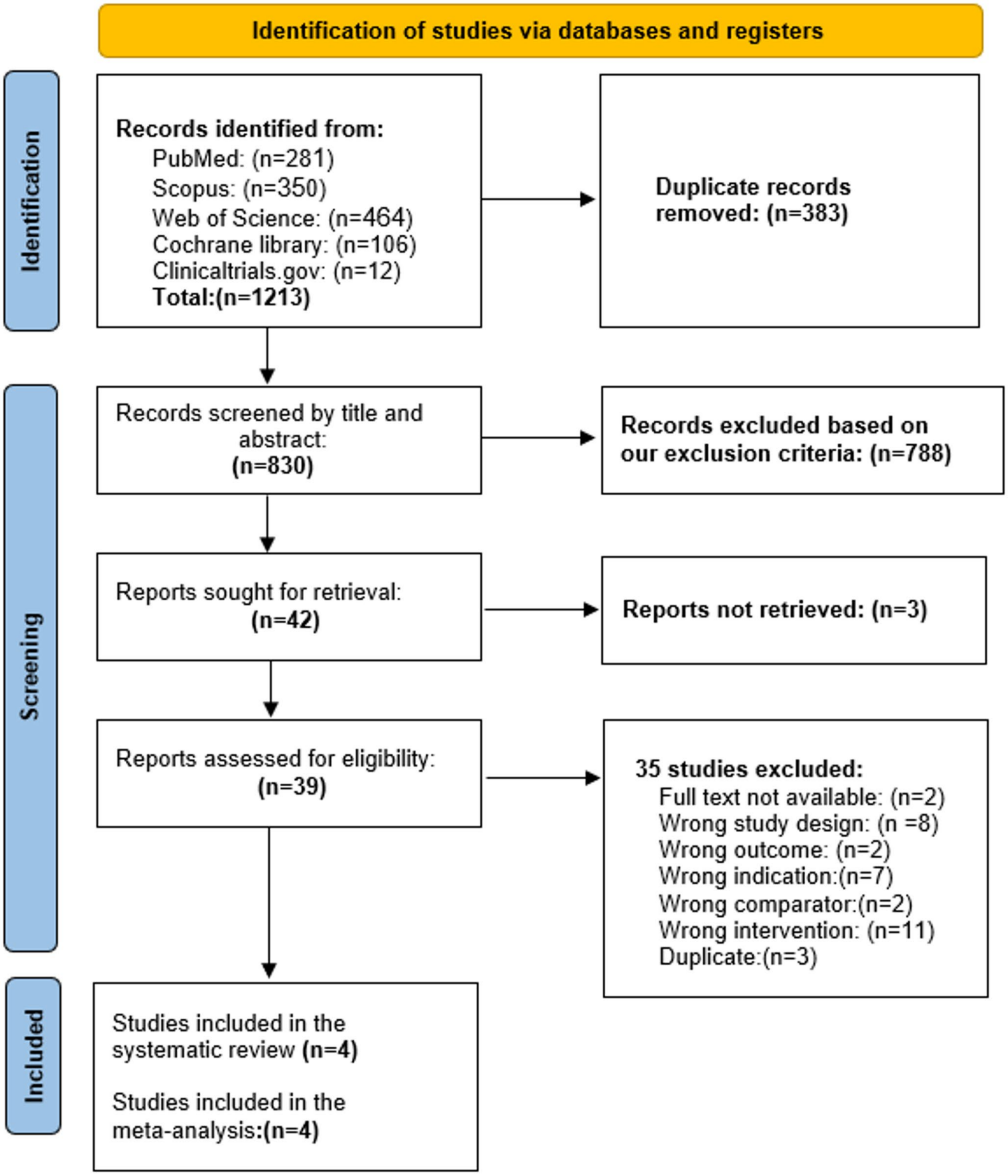
Prisma flow diagram of the included studies

A systematic search of relevant databases identified a total of 1213 articles. After removing duplicates, we excluded 830 studies during the initial screening of titles and abstracts as they did not fulfill the pre-defined inclusion criteria. This process resulted in 39 studies being selected for full-text review. Upon further evaluation, 35 studies were excluded for specific reasons, which are detailed in the PRISMA flow diagram Fig. 1. Consequently, 4 studies met all eligibility criteria and were included in the final meta-analysis.

### Summary and baseline characteristics

This meta-analysis includes four RCTs conducted in China from 2017 to 2023, which evaluated the efficacy and safety of normobaric hyperoxia as an adjunctive to endovascular treatment of large-vessel occlusion in the anterior circulation [23–26]. The studies involved a total of 648 ischemic stroke patients, with 349 (53.8%) participants randomly assigned to the normobaric hyperoxia plus endovascular treatment group and 299 (46.1%) to endovascular treatment alone. A summary of the included studies and baseline characteristics of these trials are shown in Tables 1 and 2. Across the four trials, the mean age ranged from 62 to 66 years, and 68.2% of patients were males. The mean NIHSS score at baseline ranged from 10 to 16. A total of 205 (31.9%) patients had a medical history of atrial fibrillation, 386 (60%) patients had hypertension, and 158 (24.6%) had diabetes. In three trials, 249 out of 543 (45.8%) patients received IV thrombolysis prior to endovascular treatment.

**Table 1:** Summary of the included studies

Study	Study design	Clinical trial registry number	Centre/Country	Duration	Sample size	Inclusion criteria	Treatment
Li 2025	RCT	NCT04681651	26 comprehensive stroke centers, China	April 2021 to Feb 2023	282	Patients aged 18–80 years with acute ischemic stroke due to large-vessel occlusion, a pre-stroke mRS score of 0 or 1, an NIHSS score of 10–18, and an ASPECTS of ≥ 6. All were eligible for endovascular treatment and could be randomized within 6 h of stroke onset.	Normobaric hyperoxia plus endovascular treatment
Li 2024	RCT	NCT05404373	Tianjin Huanhu Hospital Stroke Center, China	June 2022 to September 2023	100	Patients aged ≥ 18 years with acute anterior circulation ischemic stroke, an NIHSS score ≥ 6, and an ASPECTS ≥ 6 were included if eligible for EVT. Exclusions included rapid improvement, NIHSS < 10 with recanalization, bleeding risks, respiratory conditions, unstable vitals, or inability to complete follow-up.	Normobaric hyperoxia plus endovascular treatment
Li 2022	RCT	NCT03620370	Xuanwu Hospital of Capital Medical University in China	August 2018 to October 2019	86	Patients aged 18–80 years with suspected proximal anterior circulation occlusion, NIHSS ≥ 6, and ASPECTS 7–10 were included. Stroke onset was ≤ 6 h or 6–24 h with a mismatch volume greater than 15 mL on CT perfusion imaging. They had no significant pre-stroke disability (mRS ≤ 1), and informed consent was obtained.	Normobaric hyperoxia plus endovascular treatment
Cheng 2021	RCT	ChiCTR-INR-17,013,685	Beijing Luhe Hospital Capital Medical University, China	December 2017 to December 2019	180	The study included AIS patients aged 18–80 with anterior circulation large vessel occlusion confirmed by CTA, MRA, or DSA. Eligible patients had NIHSS scores between 6–25, ASPECT scores of 6–10, and were treated within 6 h of stroke onset.	Normobaric hyperoxia plus endovascular treatment

Study	Study design	Treatment Protocol	Comparator	Outcome measures	Follow-up	Conclusion	
Li 2025	RCT	Oxygen therapy was administered within 30 min of treatment allocation using 100% oxygen at 10 L/min via a non-rebreather mask for 4 h or with FiO₂ of 1.0 if intubated.	endovascular treatment plus sham normobaric hyperoxia	mRS at 90 days, infarct volume, mRS scores of 0–1, 0–2, and 4–6 at 90 days, NIHSS score at 24 h, 72 h, and 7 days; early neurological improvement at 24 h, successful vessel recanalization on post-procedural angiogram, recanalization of the occluded vessel at 24–48 h, arterial partial pressure of oxygen; score on Barthel Index. Safety outcomes were all-cause death, stroke-related death, serious adverse events, oxygen-related adverse events within 90 days including severe lung infection, pneumothorax, atelectasis, respiratory failure, acute respiratory distress syndrome, and cardiopulmonary arrest), malignant brain oedema, perioperative myocardial infarction, and acute heart failure, symptomatic intracranial haemorrhage and any intracranial haemorrhage within 24 h, and early neurological deterioration	at the end of oxygen therapy, and at 24 h, 72 h, 7 days, 30 days, and 90 days	At 90 days, NBHO group had better functional outcomes (median mRS 2 vs. 3; adjusted OR 1.65, *p* = 0.018) compared to the sham group. Mortality rates (10% vs. 12%) and serious adverse events (20% vs. 23%) were similar. Overall, normobaric hyperoxia improved recovery without increasing safety risks.	
Li 2024	RCT	Oxygen therapy began within 30 min of randomization, delivering 100% oxygen at 10 L/min via a storage mask. Depending on group assignment, treatment lasted 2, 4, or 6 h, with FiO₂ of 1.0 if intubated.	endovascular treatment plus low-flow oxygen	mRS at 90 days, infarct volume at 72 h, mRS scores of 0–2 at 90 days, NIHSS score at 24 h, 72 h, and 7 days, successful vessel recanalisation on post-procedural, arterial partial pressure of oxygen. Safety outcomes were all-cause death, stroke-related death, serious adverse events, pneumonia, symptomatic intracranial haemorrhage and any intracranial haemorrhage within 24 h, and early neurological deterioration.	at the end of oxygen therapy, and at 24 h, 72 h, 7 days, and 90 days	NBHO therapy effectiveness depended on duration, with 4- and 6-hour treatments reducing infarct volume and improving NIHSS scores compared to the control group. These groups also showed better early neurological recovery, with significant improvements at 24 h, 72 h, and 7 days, without increased safety risks. NBHO therapy combined with EVT reduced infarction volume and improved 90-day mRS scores (2 vs. 3, *p* = 0.038) compared to EVT alone. The NBHO + EVT group also had lower rates of symptomatic intracranial hemorrhage, mortality, and adverse events, though differences were not statistically significant.	
Li 2022	RCT	NBHO therapy was administered at 10 L/min using a 100% oxygen storage face mask, starting immediately after randomization in the emergency department and continuing for 4 h.	endovascular treatment	mRS at 90 days, infarct volume at 24–48 h, mRS scores of 0–2 at 90 days, NIHSS score at 24 h, 72 h, and 7 days, successful vessel recanalisation on post-procedural, arterial partial pressure of oxygen. Safety outcomes were all-cause death, stroke-related death, serious adverse events, symptomatic intracranial haemorrhage and any intracranial haemorrhage within 24 h, and early neurological deterioration.	at the end of oxygen therapy, and at 24 h, 72 h, 7 days, 30 days, and 90 days		
Cheng 2021	RCT	patients received supplemental high-flow NBHO therapy by a Venturi mask (FiO2 50%, flow 15 L/min) for 6 h	endovascular treatment plus low flow oxygen supplementation	mRS at 90 days, infarct volume at 24–48 h, mRS scores of 0–2 at 90 days, NIHSS score at 24 h, successful vessel recanalisation on post-procedural, arterial partial pressure of oxygen. Safety outcomes were symptomatic intracranial haemorrhage, pneumonia, and fatal intracranial haemorrhage.	at the end of oxygen therapy, and at 24 h, 72 h, 7 days, and 90 days	High-flow normobaric oxygen therapy after endovascular recanalization improved functional outcomes, reduced mortality, and decreased infarct volumes in anterior circulation stroke patients. No significant differences in adverse events were observed between the NBHO and control groups.	

RCT: Randomized controlled trial; mRS: Modified Rankin Scale; NIHSS: National Institutes of Health Stroke Scale; ASPECTS: Alberta Stroke Program Early CT Score; NBHO: Normobaric hyperoxia; EVT: Endovascular treatment; AIS: Acute Ischemic Stroke; CTA: Computed Tomography Angiography; MRA: Magnetic Resonance Angiography; DSA: Digital Subtraction Angiography

### Risk of bias assessment

**Fig. 2:**
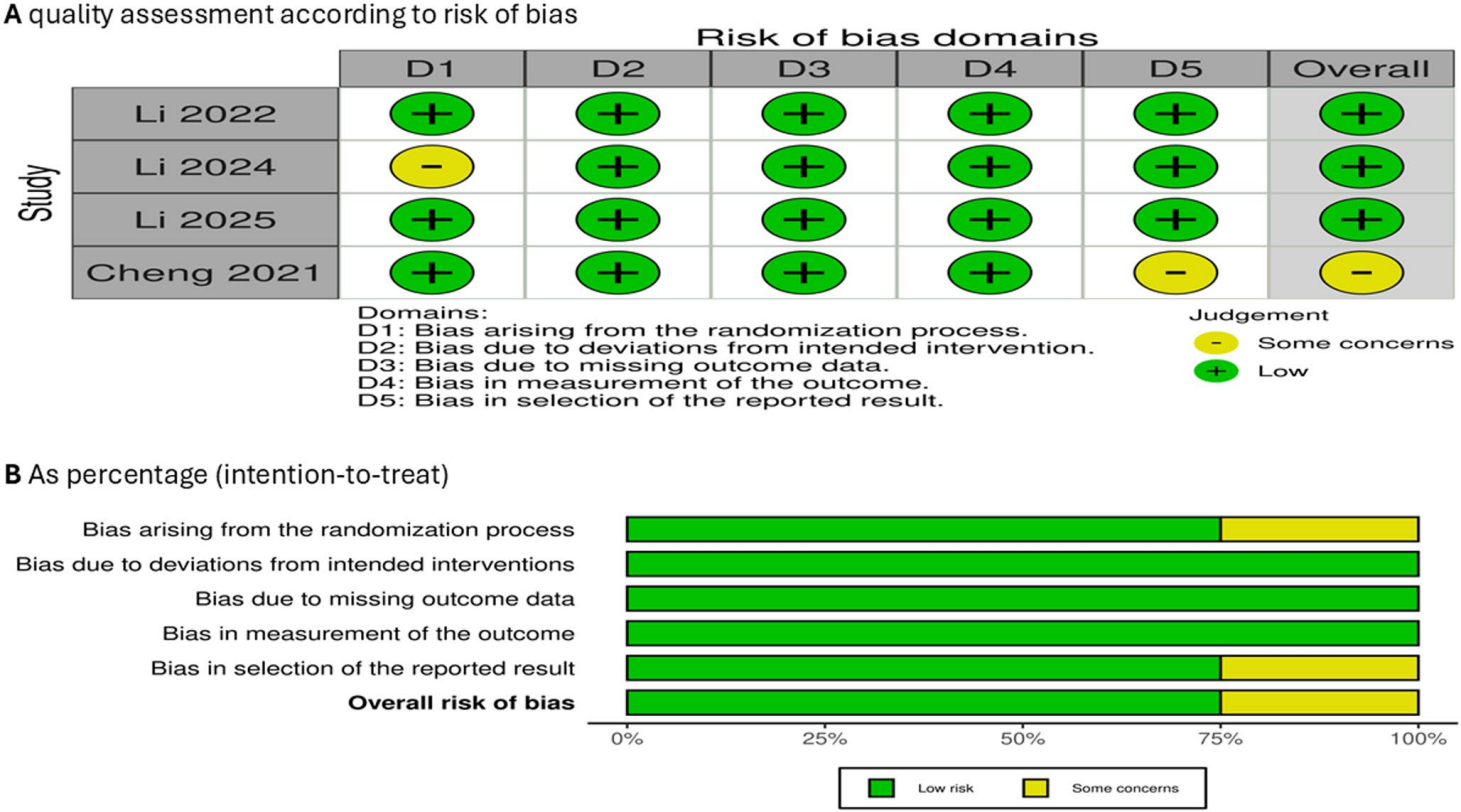
(**A**) Quality assessments according to risk of bias for each study. (**B**) Quality assessment according to risk of bias as percentage

A summary and graph of the risk of bias for the RCTs according to Cochrane risk of bias assessment tool are shown in Fig. 2. Two studies yielded a low risk of bias, while two studies demonstrated some concerns. Domain 1 (randomization process and domain 5 (Bias in selection the reported results) were the primary sources for downgrading.

**Table 2:** Baseline characteristics of the population in the included studies

Study	Group	Sample size	Age, years	Male (%)	NIHSS score	From stroke onset to NBHO therapy (hours)	Received IV thrombolysis	Atrial fibrillation	Previous ischemic stroke	Hypertension	Diabetes
Li 2025	NBHO group	140	64 (9.74)	105 (75%)	14 (3.15)	4.13 (1.8)	61 (44%)	41 (29%)	24 (17%)	69 (49%)	28 (20%)
	Control group	142	65 (11.23)	102 (72%)	14.2 (3.6)	4.27 (1.5)	64 (45%)	53 (37%)	22 (15%)	82 (58%)	34 (24%)
Li 2024	NBHO-2 h	25	61.6 (7.9)	18 (72)	10.3 (4.72)	13.33 (5.5)	NA	4 (16%)	6 (24%)	14 (56%)	6 (24%)
	NBHO-4 h	25	62.1 (8.3)	16 (64)	10.3 (3.93)	13 (5.9)	NA	5 (20%)	4 (16%)	14 (56%)	6 (24%)
	NBHO-6 h	25	62 (9.7)	18 (72%)	11.33 (6.29)	12.83 (5.5)	NA	5 (20%)	4 (16%)	16 (64%)	6 (24%)
	Control group	25	63.9 (9.6)	13 (52%)	10.67 (5.5)	12.83 (4.72)	NA	5 (20%)	5 (20%)	16 (64%)	6 (24%)
Li 2022	NBHO group	43	62 (11.7)	31 (72%)	14.33 (3.83)	4.53 (2.3)	24 (56%)	14 (33%)	12 (28%)	27 (64%)	15 (35%)
	Control group	43	64 (9.9)	29 (67%)	13.67 (3.07)	NA	22 (51%)	15 (35%)	11 (26%)	29 (67%)	11 (26%)
Cheng 2021	NBHO	88	63.8 (11.5)	56 (63.6%)	16.33 (3.01)	NA	37 (42%)	34 (38.6%)	NA	57 (64.8%)	26 (29.5%)
	Control group	87	65.9 (10.5)	51 (58.6%)	16.33 (3.77)	NA	41 (47.1%)	29 (33.3%)	NA	62 (71.3%)	20 (23%)

NIHSS: National Institutes of Health Stroke Scale; NBHO: Normobaric hyperoxia. All qualitative variables are represented as number (percentage), while quantitative variables are shown as mean (standard deviation)

### Results of the efficacy outcomes

#### mRS scores

**Fig. 3:**
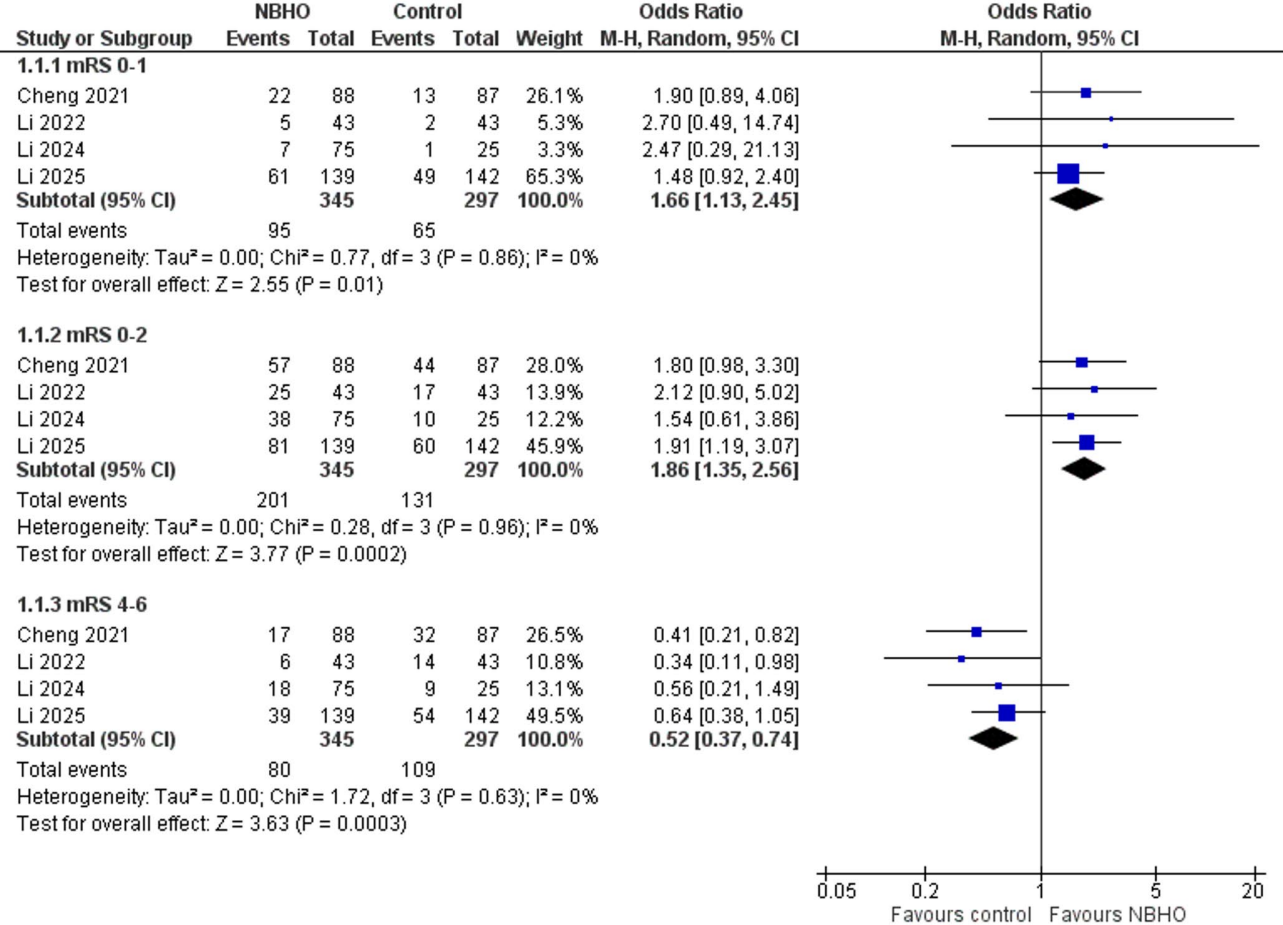
(1) mRS score between 0–1, (2) mRS score 0–2, (3) mRS score 4–6. NBHO: Normobaric hyperoxia; M-H: Mantel-Haenszel; CI: Confidence interval

**Fig. 4:**
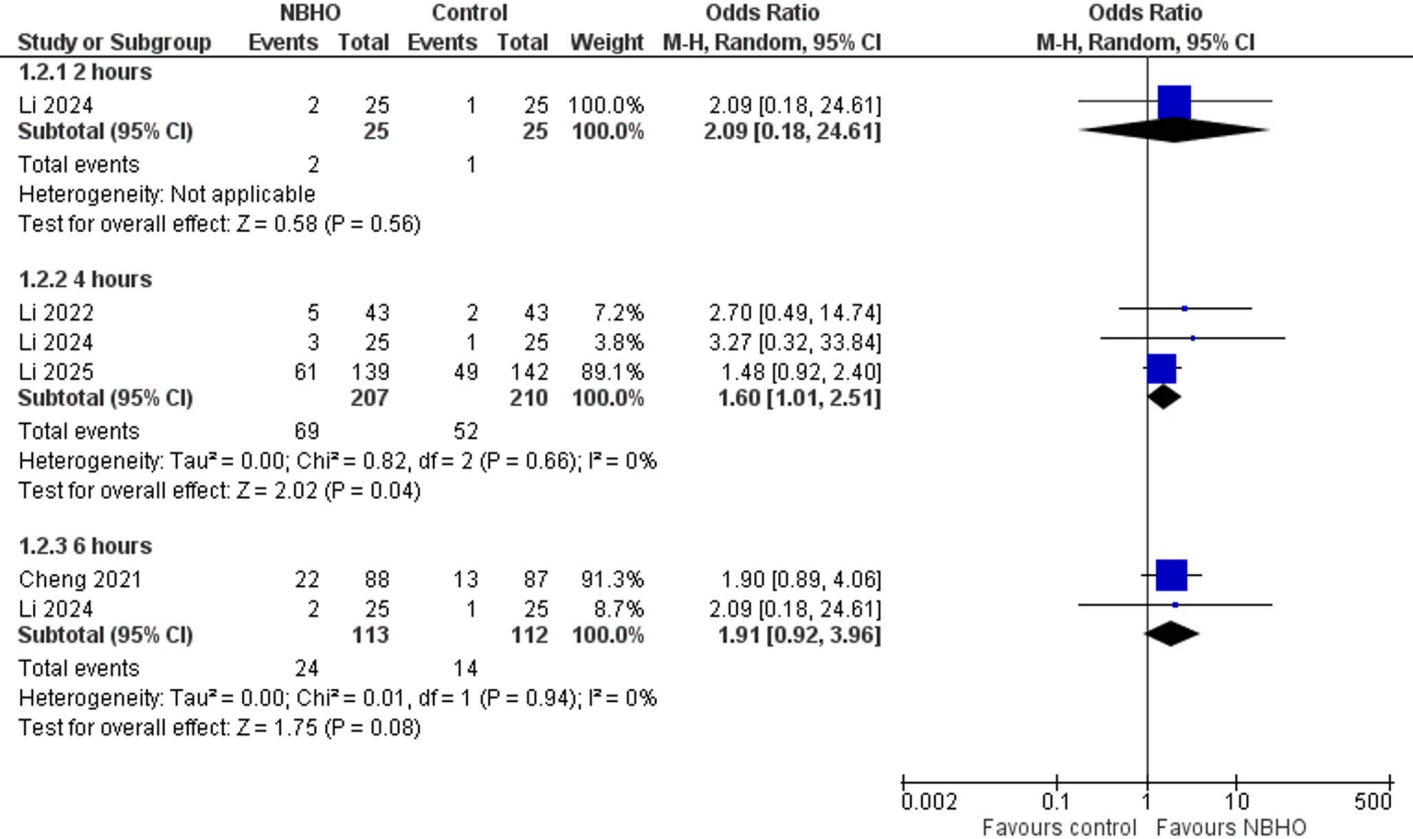
Subgroup analysis of participants who achieved mRS 0–1 according to NBHO therapy duration. NBHO: Normobaric hyperoxia; M-H: Mantel-Haenszel; CI: Confidence interval

For the primary efficacy endpoint of excellent functional outcome defined as the number of patients who had mRS score of ≤ 1 at 90 days, the overall odds ratio with subgroup based on the oxygen delivery duration at 2, 4, and 6 h was in favor of the NBHO group compared to the control (OR = 1.66, 95% CI [1.13, 2.45], *P* = 0.01, I^2^ = 0%, Fig. 3). The subgroup of 4-hour oxygen delivery duration was the only significant subgroup (OR = 1.6, 95% CI [1.01, 2.51], *P* = 0.04, I^2^ = 0% Fig. 4). Sensitivity analysis using the leave-one-out method showed consistent results (Supplementary Fig. 2). Additionally, analysis of mRS 0–2 and mRS 4–6 showed significant difference between both groups (*P* < 0.05, Fig. 3) with subgroups based on NBHO therapy duration. Both the 4-hour and 6-hour groups demonstrated a statistically significant difference, while the 2-hour group failed to show superiority over the control group, no heterogeneity was found in all the analysis (Supplementary Fig. 3,4). Three studies reported mRS scores on day 90, the overall effect was statistically significant between the two groups (MD = −0.6, 95% CI [−1.01, −0.2], *P* = 0.003, I^2^ = 17%, Supplementary Fig. 5,6).

#### NIHSS score changes from the baseline

**Fig. 5:**
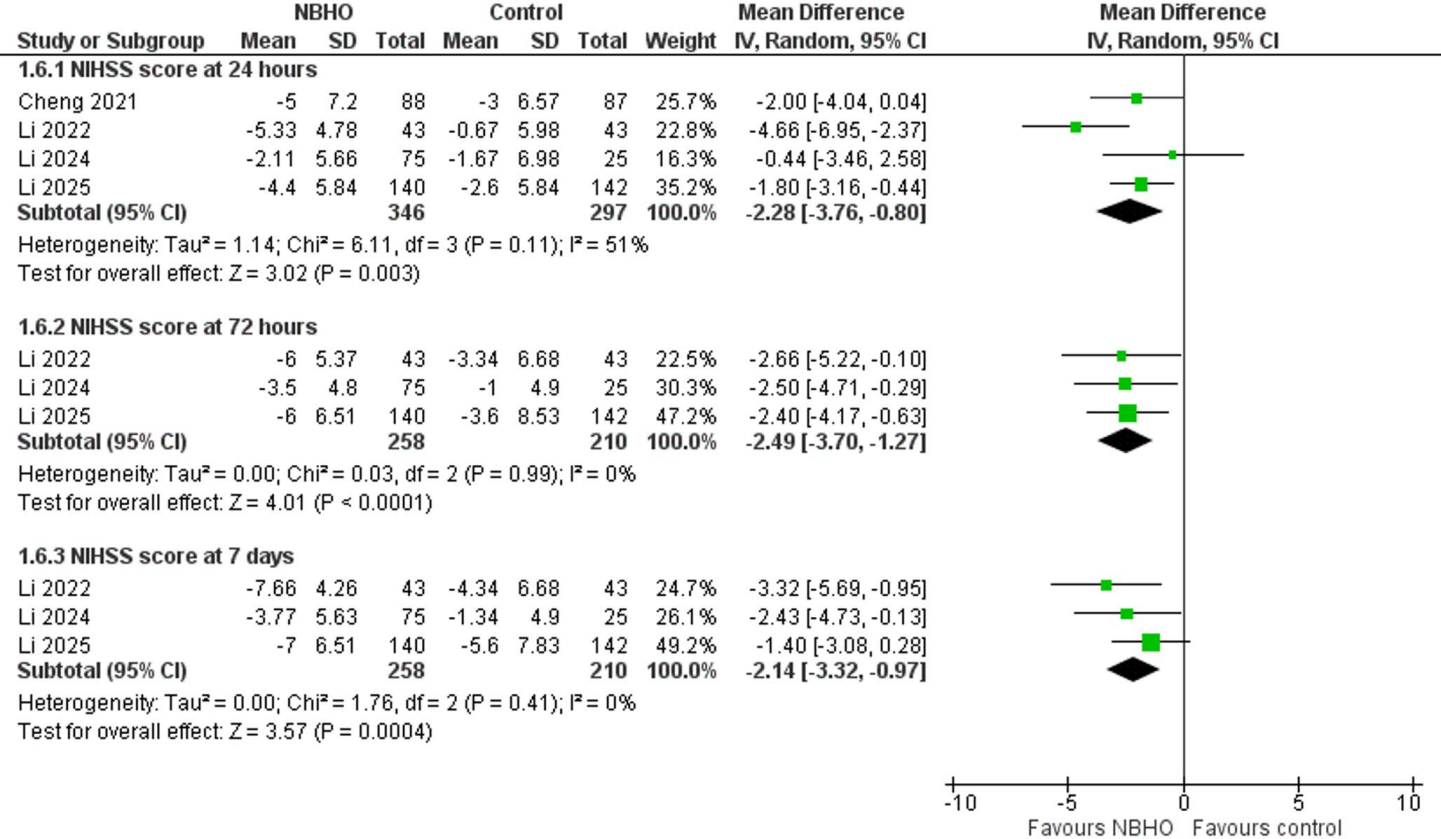
(1) NIHSS score at 24 h, (2) NIHSS score at 72 h, (3) NIHSS score at 7 days. IV: Inverse-variance, CI: Confidence interval, NBHO: Normobaric hyperoxia

For the additional efficacy endpoints including the mean change of NIHSS score from baseline, significant reduction in NIHSS score was in favor of the NBHO group compared to control at all follow-up periods (MD = −2.28, 95% CI = [−3.76, −0.80], *P* = 0.003) at 24 h, (MD = −2.49, 95% CI = [−3.70, −1.27], *P* < 0.001) at 72 h, and (MD = −2.14, 95% CI = [−3.32, −0.97], *P* = 0.004) at 7 days (Fig. 5**)**. Moderate heterogeneity was found in the 24 h subgroup (I^2^ = 51%), which was best resolved by excluding Li 2024 from the analysis. After excluding this study, the overall mean difference was still in favor of the NBHO group (MD = −1.69, 95% CI = [−2.75, −0.62], *P* = 0.002), and the heterogeneity was resolved (I^2^ = 0) (Supplementary Fig. 7). Subgroups based on NBHO therapy duration. Both the 4-hour and 6-hour groups demonstrated a statistically significant difference (Supplementary Figs. 8,9,10).

#### Infarct volume

**Fig. 6:**
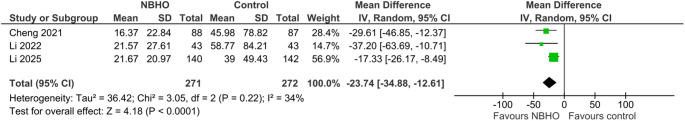
Infarct volume at 24–48 h after treatment. IV: Inverse-variance; CI: Confidence interval; NBHO: Normobaric hyperoxia

Three studies reported the endpoint of the mean infarct volume at 24–48 h after treatment, the mean infarct volume was significantly reduced in the NBHO group compared to the control group (MD = −23.74 mL, 95% CI = [−34.88, −12.61], *P* < 0.0001). No significant heterogeneity was found (I^2^ = 34%, *P* = 0.22, Fig. 6). Regarding infarct volume at 72 h, Li et al. (2024) found that 4-hour and 6-hour NBHO therapy significantly reduced infarct volume, while 2-hour treatment showed no benefit.

### Results of the safety outcomes

**Fig. 7:**
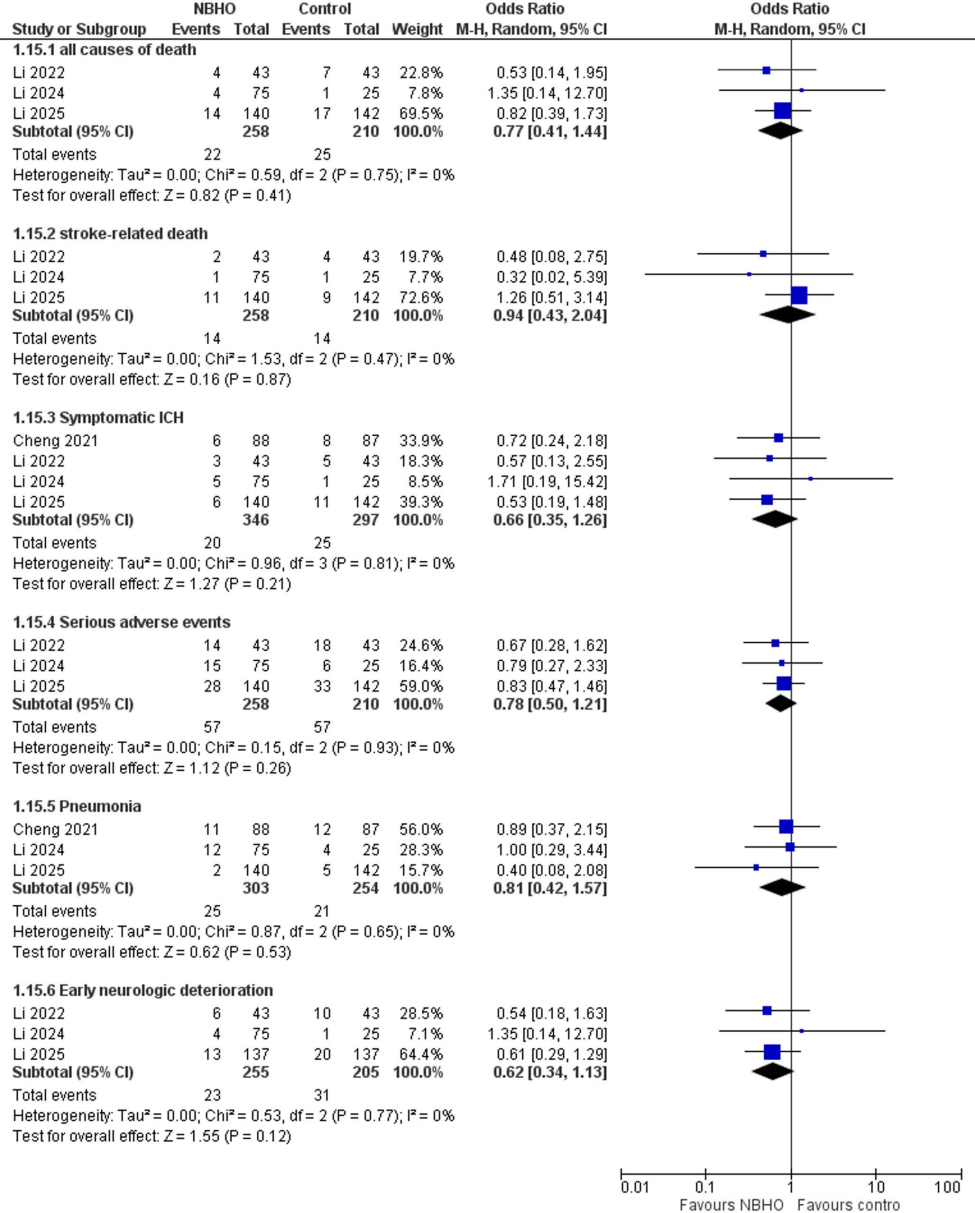
Safety outcomes analyses. NBHO: Normobaric hyperoxia; M-H: Mantel-Haenszel; CI: Confidence interval

For all safety outcomes, the analysis showed no difference between the NBHO and control groups. The pooled odds ratio was (OR = 0.77, 95% CI [0.41, 1.44], *P* = 0.41) for all-cause death outcome at 90 days, (OR = 0.94, 95% CI [0.43, 2.04], *P* = 0.87) for stroke-related deaths at 90 days, (OR = 0.66, 95% CI [0.35, 1.26], *P* = 0.87) for symptomatic intracranial hemorrhage at 24 h, for serious adverse events at 7 days (OR = 0.78, 95% CI [0.5, 1.21], *P* = 0.26), for pneumonia at 90 days (OR = 0.81, 95% CI [0.42, 1.57], *P* = 0.53), and (OR = 0.62, 95% CI [0.34, 1.13], *P* = 0.12) for early neurological deterioration outcome at 24 h. No heterogeneity was found in all safety outcomes (I^2^ = 0%) Fig. 7. Subgroup analysis according to NBHO duration showed no difference between 2-hours, 4-hours, and 6-*hours* (Supplementary Figs. 11, 12).

### GRADE assessment of the certainty in the evidence

We used the GRADE approach to assess the certainty of the evidence for efficacy outcomes, including mRS 0–1, NIHSS at 24 h, and infarct volume at 24–48 h. Moreover, we assessed safety outcomes, including symptomatic intracranial hemorrhage, all-cause mortality, stroke-related mortality, and serious adverse events. All outcomes were rated as Moderate certainty. The details of GRADE assessment are provided in Table 3.

**Table 3:** GRADE assessment of the certainty in the evidence

Certainty assessment							№ of patients		Effect size	Certainty	
№ of studies	Study design	Risk of bias	Inconsistency	Indirectness	Imprecision	Other considerations	NBHO	Control	OR or MD (95% CI)		
*mRS 0–1*											
4	RCTs	not serious	not serious	not serious	serious^**a**^	not serious	345	297	OR = 1.66 [1.13, 2.45]	⨁⨁⨁◯ Moderate	
*NIHSS score at 24 h*											
4	RCTs	not serious	serious^**b**^	not serious	not serious	not serious	346	297	MD= −2.28 [−3.76, −0.8]	⨁⨁⨁◯ Moderate	
*Infarct volume at 24–48 h*											
3	RCTs	not serious	not serious	not serious	Serious^**c**^	not serious	271	272	MD= −23.74 [−34.88, −12.61]	⨁⨁⨁◯ Moderate	
*Symptomatic Intracranial Hemorrhage*											
4	RCTs	not serious	not serious	not serious	Serious^**d**^	not serious	346	297	OR = 0.66 [0.35, 1.26]		⨁⨁⨁◯ Moderate
*All causes of death*											
3	RCTs	not serious	not serious	not serious	Serious^**d**^	not serious	258	210	OR = 0.77 [0.41, 1.44]		⨁⨁⨁◯ Moderate
*Stroke-related death*											
3	RCTs	not serious	not serious	not serious	Serious^**d**^	not serious	258	210	OR = 0.94 [0.43, 2.04]		⨁⨁⨁◯ Moderate
*Serious adverse events*											
3	RCTs	not serious	not serious	not serious	Serious^**d**^	not serious	258	210	OR = 0.78 [0.5, 1.21]		⨁⨁⨁◯ Moderate

RCT: Randomized controlled trials; OR: Odds ratio; MD: Mean difference; CI: Confidence interval; NBHO: Normobaric hyperoxia; mRS: Modified Rankin Score; NIHSS: National Institutes of Health Stroke Scale

^**a**^Downgraded due to relatively wide confidence interval and the lower bound is close to no effect

^**b**^Downgraded due to moderate heterogeneity (I^2^ = 51%)

^**c**^Downgraded due to moderate sample size and only three studies

^**d**^Downgraded as confidence intervals include both potential benefit and harm

## Discussion

Our meta-analysis provides comprehensive evidence supporting the efficacy and safety of NBHO as an adjunct EVT in AIS due to LVO. Across the four included RCTs, NBHO as an adjunct to EVT significantly improved functional outcomes in AIS. Patients receiving NBHO had a higher likelihood of achieving an excellent functional outcome (mRS ≤ 1 at 90 days, OR = 1.66, *P* = 0.01), with the 4-hour oxygen duration subgroup showing the most benefit. Additionally, NBHO resulted in greater reductions in NIHSS scores at 24 h, 72 h, and 7 days, indicating enhanced early neurological recovery. Infarct volume was significantly smaller in the NBHO group, suggesting reduced ischemic damage. The 4-hour NBHO was statistically significant in all efficacy measures, while the 6-hour NBHO showed significance in some outcome measures. In contrast, the 2-hour NBHO did not show statistical significance in any efficacy measure. There was heterogeneity in the NIHSS analysis at 24 h, which was completely resolved by excluding Li et al. (2022). This may be explained by the fact that it was the only study that used EVT alone as a comparator without sham oxygen. Importantly, NBHO did not increase the risk of symptomatic intracranial hemorrhage, mortality, or other serious adverse events, confirming its safety.

Regarding safety and feasibility, in our study, the NBHO + EVT group showed a potentially lower incidence of symptomatic intracranial hemorrhage (5.7% vs. 8.4%) and mortality (8.5% vs. 11.9%) compared to the other group. However, these differences did not reach statistical significance, likely due to the small number of cases included in the analysis. Additionally, findings from Weili Li (2022) demonstrated that NBHO therapy had no significant adverse effects on vital signs during or after administration [26]. Blood gas analysis, including pH levels, carbon dioxide partial pressure, lactic acid levels, and electrolyte balance, remained stable after oxygen therapy, further supporting the feasibility of NBHO as an adjunctive treatment. Similarly, multiple studies have reported that NBHO administration is safe in patients with acute ischemic stroke [27]. However, conflicting evidence exists in the literature, with some studies suggesting that NBHO may have harmful effects when used without EVT [28]. These concerns have led to recommendations against its routine use in non-hypoxic stroke patients with a mild to moderate stroke burden.

Previous clinical studies on NBHO therapy have employed diverse treatment protocols, yielding varying outcomes. Singhal et al. administered continuous NBHO therapy for 8 h at a high flow rate of 45 L/min using a simple face mask, reporting short-term neuroprotective benefits lasting up to one week; however, no significant effects were observed at 90 days [29]. In contrast, Padma et al. applied NBHO therapy for 12 h at a lower flow rate of 10 L/min with a simple face mask but found it to be ineffective [30]. Meanwhile, Mazdeh et al. demonstrated that NBHO therapy delivered via a Venturi mask with 50% oxygen saturation and continuous inhalation for 12 h led to improved long-term outcomes in both ischemic and hemorrhagic stroke patients (Mazdeh et al. 2015). Regarding the use of NBHO as an adjunctive to EVT, Cheng et al. used a Venturi mask (FiO₂ 50%, flow rate 15 L/min) for 6 h following vessel recanalization, concluding that high-flow NBHO therapy post-endovascular recanalization was both safe and effective, improving functional outcomes, reducing mortality rates, and minimizing infarct volumes [23]. Additionally, Li (2025) administered 100% oxygen at 10 L/min via a non-rebreather mask for 4 h, observing long-term neuroprotective effects at 90 days [25]. A similar protocol was applied in Li (2025) using a 100% oxygen storage face mask immediately after randomization in the emergency department, with treatment continuing for 4 h, yielding comparable results [26].

NBHO as an adjunct to EVT showed significant improvement in functional outcomes. This can be attributed to the pathophysiology of AIS, which results from vessel occlusion, leading to a severe reduction in oxygen supply to brain tissues. The resulting hypoxia is a key pathological event that triggers a cascade of tissue injury, primarily by reducing the interstitial partial pressure of oxygen (pO₂). While the ischemic core experiences near-zero pO₂ levels, the surrounding penumbral tissue undergoes moderate oxygen depletion, making it vulnerable to further damage. Supplementing oxygen to these ischemic regions can help preserve the penumbra, prevent infarct expansion, and potentially extend the therapeutic window for revascularization [31]. Enhanced penumbral oxygenation is believed to mitigate ischemic injury by reducing the production of reactive oxygen species (ROS), matrix metalloproteinase-9 (MMP-9), and caspase-8, thereby minimizing the no-reflow phenomenon and limiting secondary damage [32]. Additionally, oxygen easily diffuses across the blood–brain barrier (BBB), offering the potential to counteract hypoxia-induced brain injury and improve clinical outcomes [33].

However, previous studies have failed to demonstrate a definitive neuroprotective benefit of NBHO therapy in stroke patients. It is important to note that these clinical trials did not incorporate endovascular recanalization, which may have influenced the overall effectiveness of NBHO as an adjunctive treatment [34, 35].

### Limitations and future research

This meta-analysis has several limitations that should be acknowledged. First, the number of included RCTs was limited, with all studies conducted in China, potentially affecting the generalizability of our findings to diverse populations with different healthcare systems and stroke management protocols. Second, the included studies had relatively small sample sizes, limiting statistical power to detect rare adverse events. Third, variations in NBHO administration protocols, including differences in oxygen flow rates and durations, introduce heterogeneity that may influence outcomes. Although we performed subgroup analyses, further standardization of NBHO protocols is needed. Our subgroup findings should be regarded as exploratory and hypothesis-generating and interpreted with caution. Additionally, outcome assessments such as NIHSS and mRS scores were subject to inter-rater variability despite being widely used. Lastly, long-term outcomes beyond 90 days were not assessed, limiting our understanding of NBHO’s sustained benefits.

Future research should focus on large-scale, multicenter RCTs across different regions to validate our findings and enhance external validity. Additionally, studies with long-term follow-up were needed to better evaluate the potential risks associated with prolonged oxygen exposure. Further studies should explore optimal NBHO administration parameters, including timing, duration, and flow rate, to maximize neuroprotection. Additionally, investigating the underlying mechanisms of NBHO’s neuroprotective effects, particularly its impact on oxidative stress and inflammatory pathways, may provide more profound insights into its therapeutic potential in acute ischemic stroke.

## Conclusion

The result suggests NBHO significantly improved functional outcomes, reduced infarct volume, and enhanced early neurological recovery without increasing safety risks in anterior circulation LVO stroke patients. Notably, a 4-hour oxygen administration duration yielded the most favorable results. While our findings highlight NBHO as a promising neuroprotective strategy. Further RCTs are needed to confirm our results and to establish the optimal treatment protocol.

## Supplementary Information

Below is the link to the electronic supplementary material.

Supplementary Material 1

## Data Availability

The data that support the findings of this study are available from the corresponding author upon reasonable request.
