# Supplementary material for: Efficacy and safety of normobaric hyperoxia as an adjunct to endovascular thrombectomy in acute ischemic stroke: A systematic review and meta-analysis of randomized controlled trials
Source: J Thromb Thrombolysis. 2025 Nov 8;59(3):800–14. doi: 10.1007/s11239-025-03193-0 (PMC13246898; doi:10.1007/s11239-025-03193-0)
Supplement: Supplementary file 1 — Supplementary Material 1 [file 11239_2025_3193_MOESM1_ESM.docx]

| **Appendix A**: databases were used to search for articles related to the following key words: | | |
| --- | --- | --- |
| Number | Search strategy | Database |
| 281 | ("normobaric hyperoxia" OR "high-flow oxygen" OR "high oxygen therapy" OR "100% oxygen") AND ("acute ischemic stroke" OR "ischemic stroke" OR "stroke" OR "cerebral ischemia" OR "brain ischemia" OR "cerebral infarction")) | PubMed |
| 350 | TITLE-ABS-KEY (("normobaric hyperoxia" OR "high-flow oxygen" OR "high oxygen therapy" OR "100% oxygen")) AND TITLE-ABS-KEY (("acute ischemic stroke" OR "ischemic stroke" OR "stroke" OR "cerebral ischemia" OR "brain ischemia" OR "cerebral infarction")))) | Scopus |
| 464 | ALL=(("normobaric hyperoxia" OR "high-flow oxygen" OR "high oxygen therapy" OR "100% oxygen"))AND ALL=(("acute ischemic stroke" OR "ischemic stroke" OR "stroke" OR "cerebral ischemia" OR "brain ischemia" OR "cerebral infarction")))) | Web of science |
| 106 | ("normobaric hyperoxia" OR "high-flow oxygen" OR "high oxygen therapy" OR "100% oxygen")  AND  ("acute ischemic stroke" OR "ischemic stroke" OR "stroke" OR "cerebral ischemia" OR "brain ischemia" OR "cerebral infarction") | Cochrane library |
| 12 | (Normobaric hyperoxia" OR high-flow oxygen)  AND  ischemic stroke OR brain ischemia)) | Clinicaltrials.gov |
| 1213 | The total from all four databases: | |
| 383 | Number of duplicates detected by (End Note): | |
| 830 | Number after deleting duplicates: | |

**Supplementary Table 1:** Search strategies and results for each database.


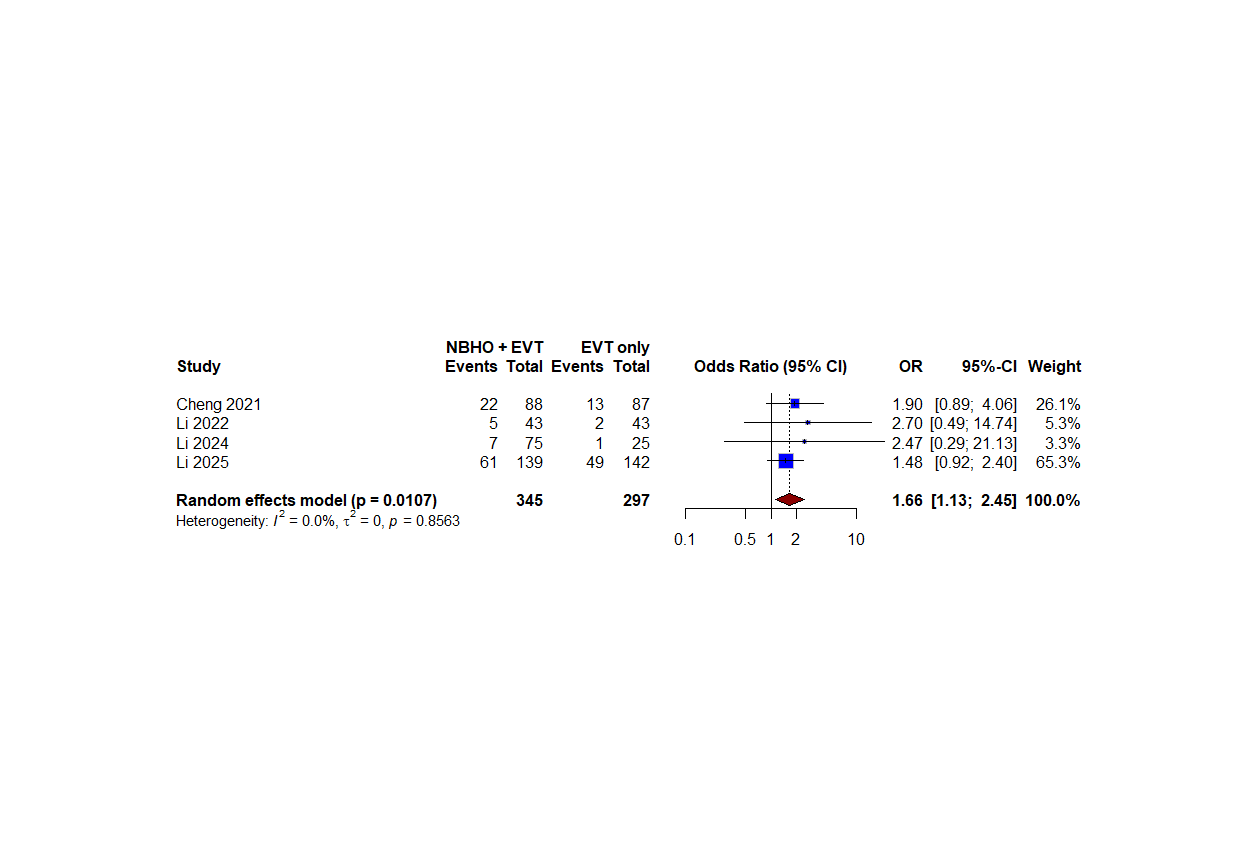
 **Figure 1:** Sensitivity analysis for mRS 0–1 at 90 days using Hartung-Knapp adjustment for the random-effects model. NBHO: Normobaric hyperoxia; EVT: Endovascular thrombectomy; OR: Odds ratio; CI: Confidence interval.

:


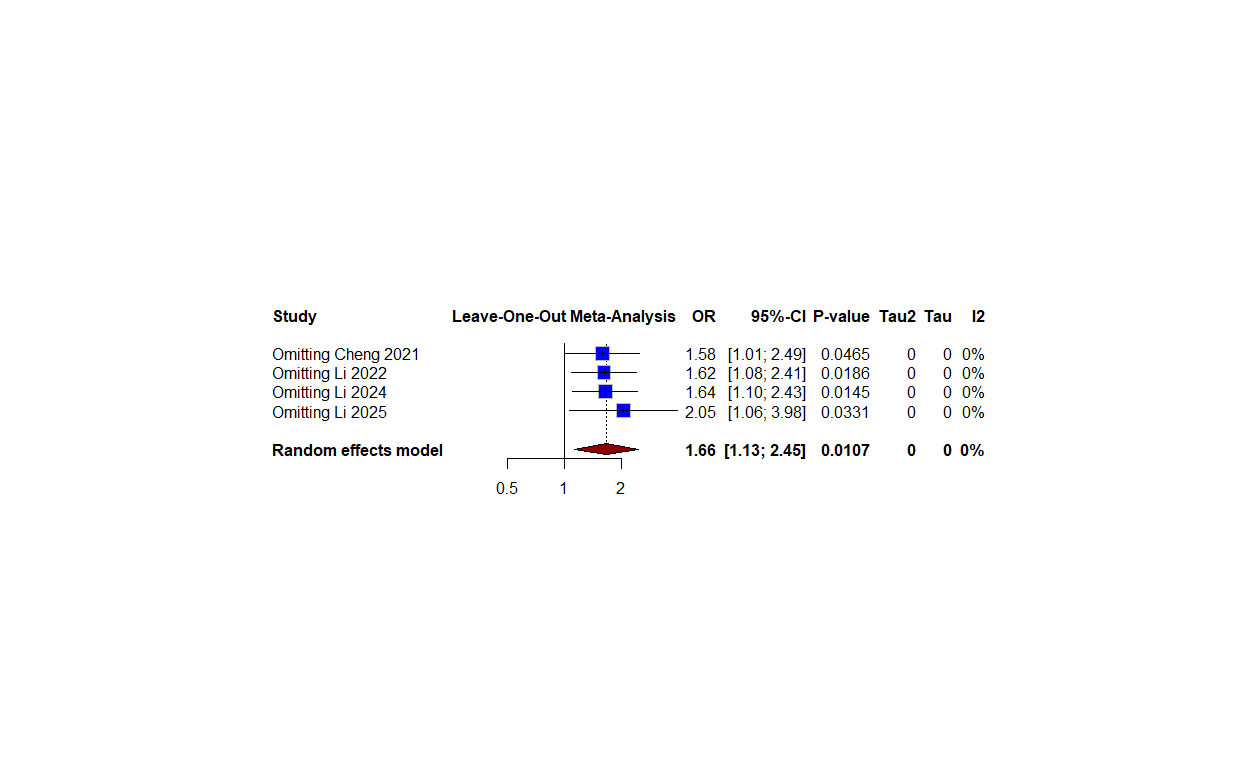

**Figure 2:** Leave-one-out sensitivity analysis for mRS 0–1 at 90 days using a random-effects model. OR: Odds ratio; CI: Confidence interval.


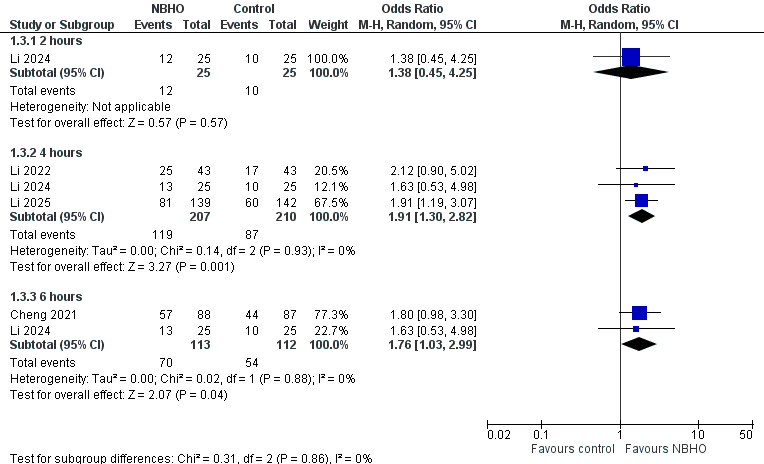


**Figure 3:** Subgroup analysis of participants who achieved mRS 0-2 according to NBHO therapy duration. NBHO: Normobaric hyperoxia; M-H: Mantel-Haenszel; CI: Confidence interval.


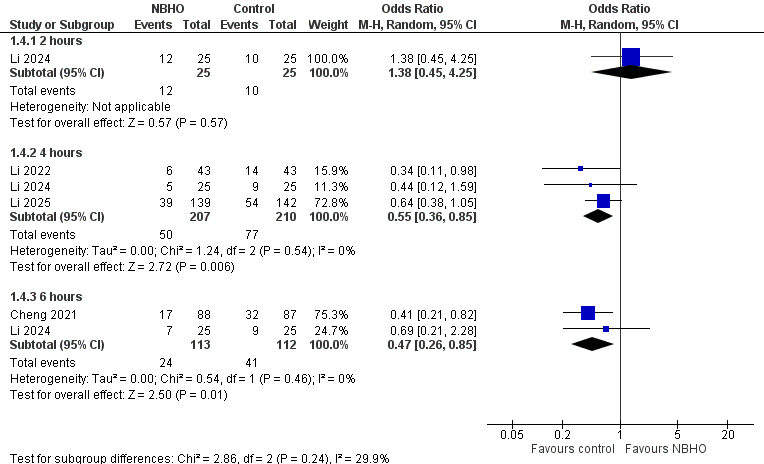


**Figure 4:** Subgroup analysis of participants who achieved mRS 4-6 according to NBHO therapy duration. NBHO: Normobaric hyperoxia; M-H: Mantel-Haenszel; CI: Confidence interval.


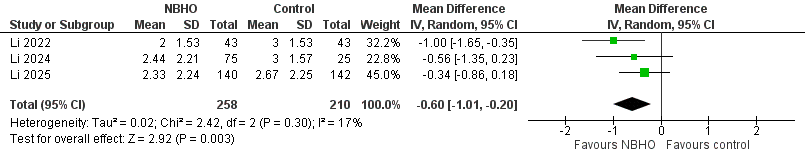


**Figure 5:** mRS score analysis at 90 days. IV: Inverse-variance; CI: Confidence interval; NBHO: Normobaric hyperoxia.


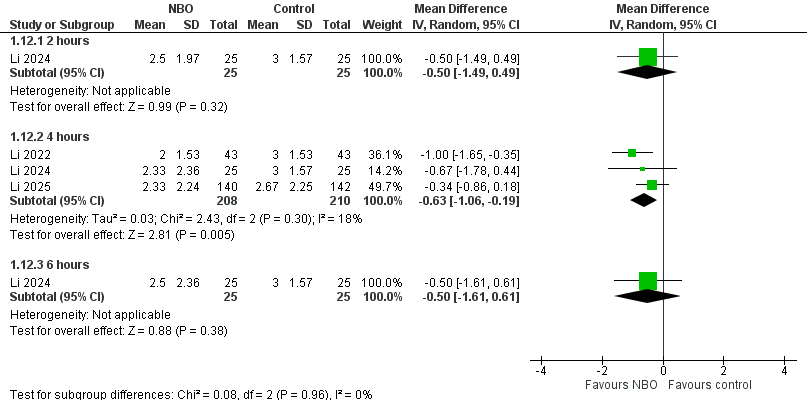
**Figure 6:** Subgroup analysis of mRS score at 90 days according to NBHO therapy duration. IV: Inverse-variance; CI: Confidence interval; NBHO: Normobaric hyperoxia.

**
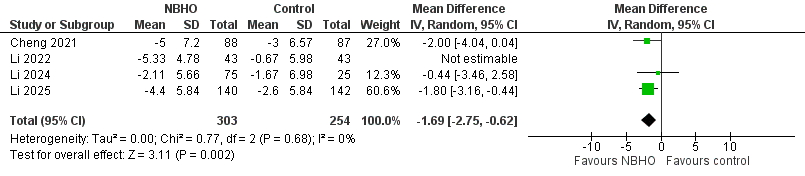
Figure 7:** Sensitivity analysis of NIHSS score at 24 hours. IV: Inverse-variance; CI: Confidence interval; NBHO: Normobaric hyperoxia.


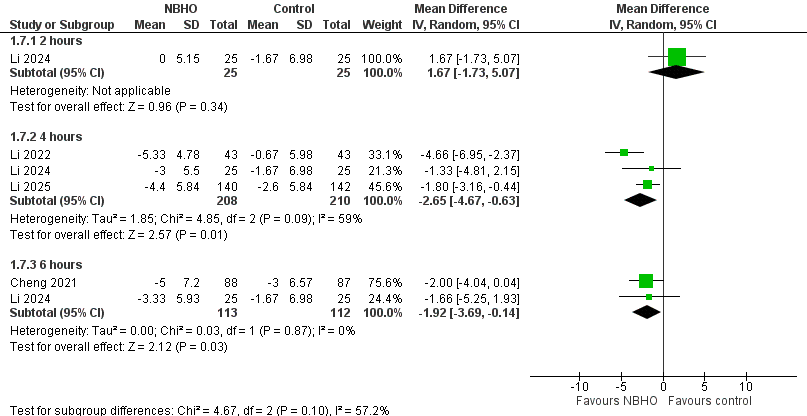
**Figure 8:** Subgroup analysis of NIHSS at 24 hours according to NBHO therapy duration. IV: Inverse-variance; CI: Confidence interval; NBHO: Normobaric hyperoxia.


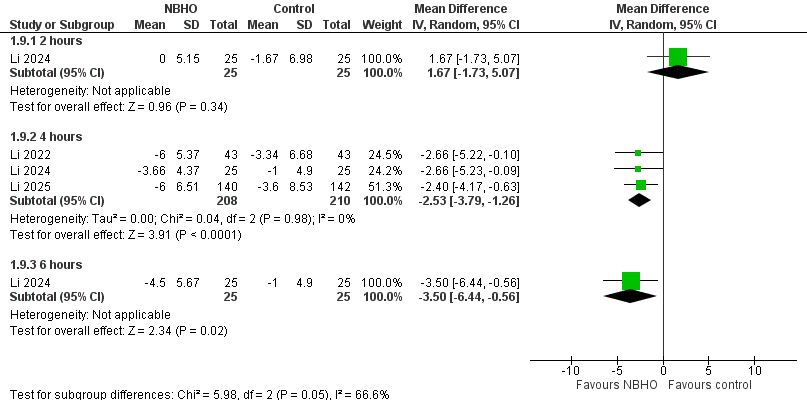
**Figure 9:** Subgroup analysis of NIHSS at 72 hours according to NBHO therapy duration. IV: Inverse-variance; CI: Confidence interval; NBHO: Normobaric hyperoxia.


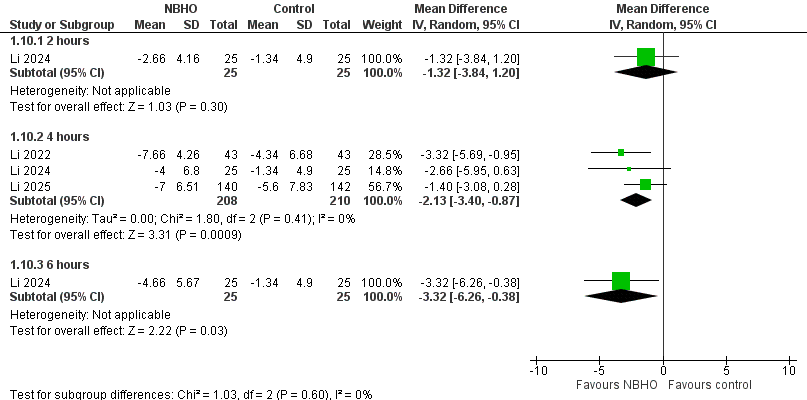
**Figure 10:** Subgroup analysis of NIHSS at 7 days according to NBHO therapy duration. IV: Inverse-variance; CI: Confidence interval; NBHO: Normobaric hyperoxia.

**
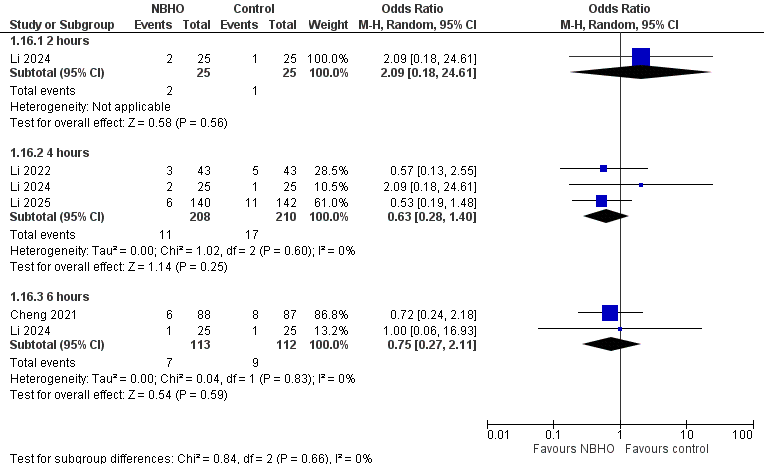
**

**Figure 11:** Subgroup analysis of symptomatic intracranial hemorrhage according to NBHO therapy duration. NBHO: Normobaric hyperoxia; M-H: Mantel-Haenszel; CI: Confidence interval.


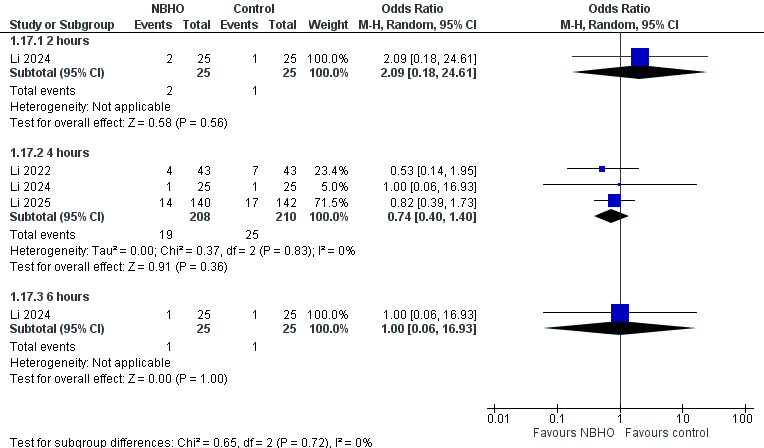
 **Figure 12:** Subgroup analysis of all causes of death at 90 days according to NBHO therapy duration. NBHO: Normobaric hyperoxia; M-H: Mantel-Haenszel; CI: Confidence interval.
